# Ambulance use is not associated with patient acuity after road traffic collisions: a cross-sectional study from Addis Ababa, Ethiopia

**DOI:** 10.1186/s12873-018-0158-5

**Published:** 2018-02-13

**Authors:** Yonas Abebe, Tolesa Dida, Engida Yisma, David M. Silvestri

**Affiliations:** 1 Department of Emergency and Critical Care Nursing, St. Paul’s Hospital Millennium Medical College, Addis Ababa, Ethiopia; 2 School of Allied Health Sciences, College of Health Sciences, Addis Ababa University, Addis Ababa, Ethiopia; 3 Robinson Research Institute, School of Medicine, The University of Adelaide, Adelaide, Australia; 4 National Clinician Scholars Program and Department of Emergency Medicine, Yale School of Medicine, New Haven, USA

**Keywords:** Ethiopia, Africa, Trauma, Road traffic collisions, Road traffic accident, Road traffic injury, Ambulance, Pre-hospital, Triage acuity, Referral

## Abstract

**Background:**

Africa accounts for one sixth of global road traffic deaths—most in the pre-hospital setting. Ambulance transport is expensive relative to other modes of pre-hospital transport, but has advantages in time-sensitive, high-acuity scenarios. Many countries, including Ethiopia, are expanding ambulance fleets, but clinical characteristics of patients using ambulances remain ill-defined.

**Methods:**

This is a cross-sectional study of 662 road traffic collisions (RTC) patients arriving to a single trauma referral center in Addis Ababa, Ethiopia, over 7 months. Emergency Department triage records were used to abstract clinical and arrival characteristics, including acuity. The outcome of interest was ambulance arrival. Secondary outcomes of interest were inter-facility referral and referral communication. Descriptive and multivariable statistics were computed to identify factors independently associated with outcomes.

**Results:**

Over half of patients arrived with either high (13.1%) or moderate (42.2%) acuity. Over half (59.0%) arrived by ambulance, and nearly two thirds (65.9%) were referred. Among referred patients, inter-facility communication was poor (57.7%). Patients with high acuity were most likely to be referred (aOR 2.20, 95%CI 1.16–4.17), but were not more likely to receive ambulance transport (aOR 1.56, 95%CI 0.86–2.84) or inter-facility referral communication (aOR 0.98, 95%CI 0.49–1.94) than those with low acuity. Nearly half (40.2%) of all patients were referred by ambulance despite having low acuity.

**Conclusions:**

Despite ambulance expansion in Addis Ababa, ambulance use among RTC patients remains heavily concentrated among those with low-acuity. Inter-facility referral appears a primary contributor to low-acuity ambulance use. In other contexts, similar routine ambulance monitoring may help identify low-value utilization. Regional guidelines may help direct ambulance use where most valuable, and warrant further evaluation.

**Electronic supplementary material:**

The online version of this article (10.1186/s12873-018-0158-5) contains supplementary material, which is available to authorized users.

## Background

Worldwide, road traffic collisions (RTC) are a leading cause of preventable morbidity and mortality. Each year, more than 1.2 million people die from RTC globally—one every thirty seconds—and as many as 50 million more incur non-fatal injuries [1, 2]. This burden is borne disproportionately by low- and middle-income countries (LMIC). Although Africa has just 2% of the world’s vehicles, it accounts for 16% of global road traffic deaths [1].

The World Health Organization has highlighted several key interventions for the prevention of RTC, including notably laws and regulations to improve driver, vehicle, and road safety [1]. Nevertheless, in conjunction with such prevention efforts, quick and effective emergency health systems are needed to limit resultant morbidity and mortality after RTC. This starts with rapid and appropriate pre-hospital care and transport. In LMIC, it is estimated that 80% of road traffic deaths occur before patients even reach a hospital [3]. Although much of this mortality may be attributed to rural regions, where collision speeds are often higher and transport times longer [4], pre-hospital delays remain a significant contributor to excess road traffic mortality in urban contexts as well, despite increased ambulance availability [5].

As pre-hospital systems become more widely available in LMIC, attention is needed to ensure appropriate use of limited ambulance services. Many types of pre-hospital care and transport systems have been described in low-income contexts [6]. Care by formally trained pre-hospital personnel and transport by equipped ambulances is relatively expensive and remains scarce compared to other lower-resource approaches (e.g., modified motorcycles), but has advantages in certain time-sensitive and high-acuity scenarios [6]. In sub-Saharan Africa, a minority of urban RTC patients arrive by ambulance [7, 8], but the precise populations receiving such pre-hospital care and transport remain ill-defined.

To address this important research gap, we aim to describe the characteristics of RTC patients arriving by ambulance at a single urban public trauma center in Addis Ababa, Ethiopia. Addis Ababa is the capital and largest city in Ethiopia, Africa’s second most populous nation. As elsewhere in Africa, RTC are a growing problem in Ethiopia, causing nearly 3500 fatalities annually and costing 1% of gross domestic product [1]. The distribution of injuries, interventions, and outcomes of RTC patients in Addis Ababa have been described [8]. Although many of these patients arrive from the scene, a large percent are referred from primary health centers or hospitals without specific trauma care capabilities. Understanding which patients receive ambulance transport, including those referred for higher levels of care, may help identify opportunities for targeted trauma system resource allocation.

## Methods

### Study design, setting, and population

We performed a retrospective chart review of all adult and pediatric patients arriving after RTC to Addis Ababa Burn Emergency and Trauma (AaBET) Hospital from August 22nd 2015 to March 9th 2016 (7 months). AaBET Hospital is a newly established 250-bed and 12 ICU-bed teaching and public referral hospital in Addis Ababa, Ethiopia, affiliated with St. Paul’s Hospital Millennium Medical College (SPHMMC). AaBET Hospital provides 24/7 specialty services in emergency medicine, critical care, trauma and acute care surgery, orthopedics, neurosurgery, and forensic medicine; patients presenting with complaints requiring additional specialty services (e.g., cardiology, gastroenterology) are stabilized and transferred to nearby SPHMMC.

All patients presenting to the AaBET Hospital emergency department (ED) pass through a single point of triage, where trained emergency triage nurses record essential clinical and demographic information and assign a triage acuity designation according to the South African Triage Scale (SATS) [9]. The SATS has been validated for use in diverse low-income urban adult and pediatric ED settings, [10–13] and has high reported inter-rater reliability among emergency nurses (ĸ = 0.92), [14, 15] and in trauma settings [16]. It is currently used in all public EDs in Addis Ababa. Patients are categorized into five priority levels (emergent, very urgent, urgent, routine, dead on arrival) based on clinical instability, vital signs, and presenting complaint [10].

### Data collection and analysis

Patient triage records were included for analysis if they were complete or partially complete. AaBET Hospital employs a hand-written triage and medical record system. All information was entered electronically into a standardized abstraction form by trained transcriptionists clinically trained in emergency care nursing and blinded to the intended analysis, in accordance with recommended best practice for retrospective chart reviews [17]. Subsequently, an audit was performed to ensure a low rate of data entry error.

Variables abstracted for analysis from triage records included patient age and sex, date of arrival to ED (weekday, weekend), patient origin (Addis Ababa, outside Addis), mode of arrival to ED (ambulance, taxi or private car, ambulatory or carried), triage acuity, referral status and source of referral (government hospital, health center, private institution), and whether communication had been received from referring institutions prior to patient arrival. Descriptive statistics were calculated for all variables. Triage acuity scores were grouped into three categories: low (routine), moderate (urgent), or high (very urgent, emergent, dead on arrival). Children were considered less than 13 years, in conjunction SATS validation [13]. We used simultaneous multivariable logistic regression to identify factors (selected a priori) independently associated with arrival by ambulance, the primary outcome of interest. Subsequent regressions were performed to determine factors associated with inter-facility referral and inter-facility referral communication, which were secondary outcomes of interest. Missing data were accounted for using multiple imputation, and all statistical analysis was performed using SPSS version 21.0.0 (IBM Corporation, 2012).

## Results

### Population characteristics

During the seven-month study period, a total of 2062 patients were evaluated after trauma in AaBET Hospital ED. RTC accounted for 662 patients (32%), of which all had complete or partially complete triage records and were included in analysis. Two thirds of RTC patients were male (66.2%), with a median age of 27 years (Table 1). Children less than 13 accounted for a minority (4.8%) of all RTC cases. Most patients (72.4%) were seen on weekdays, with over half originating from outside Addis Ababa city limits (60.6%). Nearly half were assigned low (routine) triage acuity upon arrival (44.7%), while 42.2% were designated as moderate and another 13.1% as high acuity—9.0% as very urgent, 3.8% as emergent, and two patients (0.3%) dead on arrival.

**Table 1:** Characteristics of patients presenting after RTC to AaBET Hospital, Addis Ababa

Characteristic	Total (*n = 662*)
Patient sex, n (%)^a^	
Female	224 *(33.8)*
Male	438 *(66.2)*
Patient age, n (%)^a^	
< 13	31 (4.8)
13–24	214 (32.9)
25–40	252 (38.8)
> 40	153 (23.5)
Patient origin, n (%)^a^	
Addis Ababa	259 *(39.4)*
Outside Addis	398 *(60.6)*
Date of Arrival, n (%)^a^	
Weekday	467 *(72.4)*
Weekend	178 *(27.6)*
Triage Acuity, n (%)^a,b,c^	
Low acuity	289 *(44.7)*
Moderate acuity	273 *(42.2)*
High acuity	85 *(13.1)*
Referral status/source, n (%)^a^	
Not referred (from scene)	211 *(34.1)*
Referred from government hospital	263 *(42.5)*
Referred from health center	126 *(20.3)*
Referred from private institution	19 *(3.1)*
Ref. communication, n (%)^a,d^	
Yes (before arrival)	224 *(57.7)*
No	164 *(42.3)*
Mode of arrival^a^	
Ambulance	366 *(59.0)*
Taxi or private car	234 *(37.7)*
Walking or being carried	20 *(3.2)*

^a^Percent of non-missing data reported. Missing data per variable as follows: sex (*n* = 0/662; 0%), age (*n* = 12/662; 1.8%), patient origin (5/662; 0.8%), date of arrival (17/662; 2.6%), triage acuity (15/662; 2.3%), referral status/source (43/662; 6.5%), referral communication (20/408; 4.9%), mode of arrival (42/662; 6.3%)

^b^South Africa Triage Scale acuity designations

^c^High acuity includes very urgent (58/647; 9.0%), Emergent (25/647; 3.8%) and Dead on arrival (2/647; 0.3%). Dead on arrival grouped within very urgent or emergent due to presumed scene/pre-hospital acuity

^d^Subset of referred patients only

### Inter-facility referral

Most RTC patients (65.9%) were referred to AaBET Hospital from outside institutions (Table 1), with government hospitals comprising the single largest source of referral (42.5% of all RTC patients and 64.5% of total RTC referrals) and outpatient health centers referring one fifth (20.3%) of all RTC patients. As might be expected, the frequency of referral differed when accounting for patient origin. Among patients arriving from Addis Ababa, over half (138/249; 55.4%) arrived from the scene without any prior referral, whereas 80% (292/365) of patients from outside Addis Ababa were referred via another institution (Table 2). In fact, independent of other characteristics, patients arriving from within Addis Ababa were nearly five times less likely to be referred than peers originating from outside the city (aOR 0.22, 95%CI 0.15–0.32). Sicker patients were independently more likely to have been referred to AaBET Hospital after RTC. Patients with high and moderate triage acuity were more likely to be referred compared to those with low acuity (High: aOR 2.20, 95%CI 1.16–4.17; Moderate: aOR 1.55, 95% 1.05–2.28). Neither patient age nor sex was associated with increased odds of referral.

**Table 2:** RTC patient factors associated with referral to AaBET Hospital, Addis Ababa

Characteristic	Referral status		Adjusted odds of referral
	Referred (*n* = 408)	Not Referred (*n* = 211)	
	*N (%)* ^a^	*N (%)* ^a^	*aOR (95%CI)*
Patient sex			
Female	138 *(33.8)*	78 *(37.0)*	1.0 *(ref)*
Male	270 *(66.2)*	133 *(63.0)*	1.11*(0.76–1.63)*
Patient age			
< 13	23 *(5.7)*	8 *(3.9)*	1.03 (0.39, 2.67)
13–24	122 (30.2)	78 (38.2)	0.62 (0.38, 1.03)
25–40	162 (40.1)	74 (36.3)	0.86 (0.53, 1.42)
> 40	97 (24.0)	44 (21.6)	1.0 (ref)
Patient origin			
Addis Ababa	111 *(27.5)*	138 *(65.4)*	0.22 *(0.15, 0.32)*
Outside Addis	292 *(72.5)*	73 *(34.6)*	1.0 *(ref)*
Date of Arrival			
Weekday	290 *(72.9)*	141 *(68.8)*	1.02 *(0.68, 1.54)*
Weekend	108 *(27.1)*	64 *(31.2)*	1.0 *(ref)*
Triage Acuity^b,c^			
Low Acuity	157 *(39.6)*	119 *(57.2)*	1.0 *(ref)*
Moderate Acuity	178 *(44.9)*	73 *(35.1)*	1.55 *(1.05, 2.28)*
High Acuity	61 *(15.4)*	16 *(7.7)*	2.20 *(1.16, 4.17)*
Referral status/source			
Not referred (from scene)	N/A	211 *(100)*	N/A
Referred from government hospital	263 *(64.5)*	E N/AEN	N/A
Referred from health center	126 *(30.9)*	NE N/A N	N/A
Referred from private institution	19 *(4.7)*	NE N/A N	N/A

^a^Percent of non-missing data reported

^b^South Africa Triage Scale acuity designations

^c^High acuity includes very urgent, emergent, and dead on arrival. Dead on arrival grouped within very urgent or emergent due to presumed scene/pre-hospital acuity

### Inter-facility communication

Despite the high frequency of RTC referrals, overall communication from referring institutions to AaBET Hospital was poor. Among all RTC patients referred from other health institutions, more than two in five (164/388; 42.3%) arrived with no prior notification from the referring facility (Table 1). Communication was most likely to occur on weekdays (aOR 1.74, 95%CI 1.06–2.85), when health facility staffing is highest. Rates of communication were similar between patients referred from within (64/104; 61.5%) and from outside (156/279; 55.9%) Addis Ababa, and did not significantly differ according to the type of referring institution. Interestingly, despite higher acuity among referred RTC patients than those not referred, communication from transferring facilities was no higher among the most ill than the least (aOR 0.98, 95%CI 0.49–1.94), nor among those sent by ambulance (aOR 1.46, 95%CI 0.88–2.43) (Table 3).

**Table 3:** RTC patient factors associated with inter-facility referral communication, AaBET Hospital, Addis Ababa

Characteristic	Referral communication		Adjusted odds of communication
	Yes (*n* = 224)	No (*n* = 164)	
	*N (%)* ^a^	*N (%)* ^a^	*aOR (95%CI)*
Patient sex			
Female	82 *(36.6)*	50 *(30.5)*	1.0 *(ref)*
Male	142 *(63.4)*	114 *(69.5)*	0.69 *(0.43, 1.12)*
Patient age			
< 13	11 *(5.0)*	12 *(7.3)*	0.73 *(0.27, 1.97)*
13–24	66 (30.0)	50 (30.5)	0.98 *(0.54, 1.79)*
25–40	93 (42.3)	61 (37.2)	1.38 (0.78, 2.45)
> 40	50 (22.7)	41 (25.0)	1.0 *(ref)*
Patient origin			
Addis Ababa	64 *(29.1)*	40 *(24.5)*	1.32 *(0.79, 2.20)*
Outside Addis	156 *(70.9)*	123 *(75.5)*	1.0 *(ref)*
Date of Arrival			
Weekday	171 *(77.7)*	104 *(65.4)*	1.74 *(1.06, 2.85)*
Weekend	49 *(22.3)*	55 *(34.6)*	1.0 *(ref)*
Triage Acuity^b,c^			
Low Acuity	85 *(38.8)*	61 *(38.9)*	1.0 *(ref)*
Moderate Acuity	102 *(46.6)*	69 *(43.9)*	1.11 *(0.68, 1.79)*
High Acuity	32 *(14.6)*	27 *(17.2)*	0.98 *(0.49, 1.94)*
Referral source			
Referred from government hospital	130 *(58.0)*	121 *(73.8)*	0.55 *(0.18, 1.68)*
Referred from health center	80 *(35.7)*	38 *(23.2)*	1.15 *(0.36, 3.72)*
Referred from private institution	14 *(6.2)*	5 *(3.0)*	0 *(ref)*
Mode of arrival (transport)			
Ambulance	136 *(66.0)*	121 (77.6)	0 *(ref)*
No ambulance	70 *(34.0)*	35 (22.4)	1.46 *(0.88, 2.43)*

^a^Percent of non-missing data reported

^b^South Africa Triage Scale acuity designations

^c^High acuity includes very urgent, emergent, and dead on arrival. Dead on arrival grouped within very urgent or emergent due to presumed scene/pre-hospital acuity

### Mode of arrival

The vast majority (96.7%) of RTC patients arrived at AaBET hospital by vehicle, including over half (59.0%) by ambulance (Table 1). Regardless of other characteristics, patients were more likely to arrive by ambulance if coming from another government hospital than from the scene itself (aOR 4.23, 95%CI 2.63–6.78; Table 4). However, ambulance use did not differ significantly based on the day of patients’ arrival, nor whether they originated from within or outside Addis Ababa. Importantly, patients with highest acuity were not more likely to arrive by ambulance than those with lowest severity injuries (aOR 1.56, 95%CI 0.86–2.84). Interestingly, males were less likely to arrive by ambulance after RTC than their female counterparts (aOR 0.63, 95%CI 0.43–0.91), independent of other factors.

**Table 4:** RTC patient factors associated with ambulance arrival, AaBET Hospital, Addis Ababa

Characteristic	Arrival by ambulance		Adjusted odds of ambulance use
	Yes (*n* = 366)	No (*n* = 254)	
	*N (%)* ^a^	*N (%)* ^a^	*aOR (95%CI)*
Patient sex			
Female	139 *(38.0)*	74 *(29.1)*	1.0 *(ref)*
Male	227 *(62.0)*	180 *(70.9)*	0.63 (0.43, 0.91)
Patient age			
< 13	19 (5.3)	12 (4.8)	0.77 (0.32,1.86)
13–24	115 (31.9)	83 (33.3)	1.21 (0.75, 1.95)
25–40	145 (40.2)	93 (37.3)	1.15 (0.73, 1.82)
> 40	82 (22.7)	61 (24.5)	1.0 *(ref)*
Patient origin			
Addis Ababa	121 *(33.4)*	119 *(46.9)*	0.78 (0.53, 1.13)
Outside Addis	241 *(66.6)*	135 *(53.1)*	1.0 *(ref)*
Date of Arrival			
Weekday	257 *(72.6)*	177 *(70.8)*	1.05 (0.71, 1.55)
Weekend	97 *(27.4)*	73 *(29.2)*	1.0 *(ref)*
Triage Acuity^b,c^			
Low Acuity	145 *(41.1)*	132 *(52.2)*	1.0 *(ref)*
Moderate Acuity	155 *(43.9)*	97 *(38.3)*	1.34 (0.92, 1.95)
High Acuity	53 *(15.0)*	24 *(9.5)*	1.56 (0.86, 2.84)
Referral status/source			
Referred from government hospital	191 *(54.6)*	55 *(23.9)*	4.23 (2.63, 6.78)
Referred from health center	68 *(19.4)*	51 *(22.2)*	1.61 (0.97, 2.69)
Referred from private institution	7 *(2.0)*	9 *(3.9)*	0.88 (0.29, 2.69)
Self-Referral/Scene	84 *(24.0)*	115 *(50.0)*	1.0 *(ref)*

^a^Percent of non-missing data reported

^b^South Africa Triage Scale acuity designations

^c^High acuity includes very urgent, emergent, and dead on arrival. Dead on arrival grouped within very urgent or emergent due to presumed scene/pre-hospital acuity

## Discussion

Road traffic collisions are a leading cause of morbidity and mortality worldwide and particularly in sub-Saharan Africa, where growth in motorization has outpaced efforts to implement and enforce RTC prevention interventions [1]. Estimated annual African RTC fatality rates (26 per 100,000 population) are twice those in the United States and three times greater than in Europe [1]. In Ethiopia, RTC fatality rates are among the continent’s highest [18].

The alarming burden of RTC has attracted mounting international attention in recent years. The United Nations General Assembly declared 2011–2020 the Decade of Action for road safety [2], while the body’s Sustainable Development Goals (SDGs), ratified unanimously in 2015, called for 50% fewer RTC deaths and injuries by 2020 [19]. A number of national and local policy efforts have been implemented toward this end [20, 21]. These have mostly included legislative regulations, educational interventions, law enforcement, infrastructural reform, and community-based prevention programs [21]. At the same time, a growing number of sub-Saharan African trauma registries have aimed to provide clinicians, researchers, and administrators clearer data regarding local burden of traumatic illness [18, 22, 23].

Despite positive trends in growing global attention to RTC, surprisingly little focus has been placed on evaluating and improving formal pre-hospital trauma systems in low-income settings. Most trauma deaths occur in the pre-hospital setting, and the percent who die before reaching a hospital is highest in low-income settings where formal pre-hospital systems are weakest [24]. Most studies evaluating ambulance use after RTC have described the frequency of use [25–29], rather than independent predictors of use. Knowing which patients currently receive ambulance transport after RTC is essential to developing policies to direct its use given relative scarcity and cost compared to other modes of transport.

In our study of over six hundred RTC patients arriving to a single tertiary trauma referral hospital in Addis Ababa, Ethiopia, a higher percentage of patients arrived by ambulance (59%) compared to other published studies from elsewhere across Africa [7, 8, 25–27]. Such disparities in ambulance utilization are likely multifactorial. Our methodological decision to focus exclusively on RTC rather than all trauma patients resulted in our exclusion of large numbers of violent trauma victims transported by police, accounted for in other studies [7]. In addition, Ethiopian governmental efforts to coordinate and concentrate specialty care across city public hospitals in Addis Ababa means AaBET Hospital is one of only two major trauma centers citywide, receiving a higher proportion of regional trauma referrals than reported elsewhere [25–27]. Finally, as one of the first African countries to prioritize emergency care specialization and specialty training [28–30], Ethiopia has also placed comparatively large emphasis on formal pre-hospital infrastructure and training. Large ambulance provisions by the federal government have supplemented private and non-governmental pre-hospital organizations already in practice [31], while a regional emergency medical dispatch phone line enables easy public ambulance access. These factors may have contributed to increased ambulance availability.

Even accounting for higher rates of ambulance use, our findings highlight several important yet concerning trends that warrant further attention in other settings. Importantly, we found that ambulance use among RTC patients arriving to AaBET Hospital was not significantly associated with triage acuity. This is in contrast to prior work among both trauma and non-trauma patients in Ghana [32], and suggests that ambulance adoption in lower-income urban settings may not always naturally align with patient clinical needs. RTC patients with high acuity need time-sensitive stabilization and are at greatest risk of mortality, disability, or hospital admission, yet in our population were no more likely to receive ambulance transport than those with minimal injury burden. There are several possible explanations for this. While these findings may reflect persistent pervasive public unawareness regarding ambulance services or public ambulance avoidance due to fear of financial liability, we instead suspect they represent a discrepancy in the extent to which patient acuity influences ambulance selection among referred and non-referred populations.

To better test this hypothesis, we performed separate subgroup analyses assessing ambulance use among referred and non-referred populations (i.e., effect modification). Results did not change among patients referred to AaBET from another institution; patients with high and moderate acuity were no more likely to be transported by ambulance than those with low acuity (Additional file 1: Table S1). Similarly, among patients arriving directly from the scene, those with high acuity were not more likely to receive ambulance transport than those with low acuity (Additional file 2: Table S2). However, those with moderate acuity from the scene were nearly three times more likely to arrive by ambulance than those with low acuity (aOR 2.97, 95%CI 1.50–5.86). This finding is important, and suggests that transportation decisions made at the scene may be influenced by patient acuity. In light of this, lower rates of ambulance transportation among high-acuity patients at the scene may reflect lay first responders’ preferential selection of immediate on-site (lay) transportation for the sickest patients deemed unable to wait for ambulance arrival. By contrast, we found no evidence to suggest that acuity influenced ambulance decisions made by referring providers—consistent as well with poor inter-facility communication (58%), even for the sickest patients.

In low-income countries, ambulances remain scare resources but possess key benefits over pre-hospital lay transportation in the appropriate clinical settings. Given the relative expense of ambulances in such settings, maximizing their value is essential, and will depend on judicious use in accordance with patient clinical needs. When assessed alongside contrasting work from Ghana [32], our findings suggest heterogeneity in post-traumatic ambulance utilization patterns in sub-Saharan Africa, and highlight the need for locally-driven assessments to ensure value-directed care. Furthermore, our data suggest that in promoting appropriate ambulance use, training of healthcare providers may be equally important as ongoing lay education. Inappropriate inter-facility ambulance use for patients deemed low-acuity after clinician evaluation offers few benefits over lay transportation, while limiting ambulance availability in higher-acuity settings elsewhere. When ambulances are free to respond in a timely fashion to high-acuity patients, a greater proportion of these patients may be brought directly from the scene to trauma centers like AaBET Hospital, bypassing outside institutions with little or no trauma expertise and improving mortality [33]. In our study, 103 low-acuity patients received ambulance transport from another facility (40% of all referred patients transported by ambulance), while one third (40) of high- and moderate-acuity patients arrived from the scene by other means (Additional file 1: Table S1 and Additional file 2: Table S2).

We suggest that countries like Ethiopia that seek to develop formal pre-hospital ambulance transportation systems should develop measures to ensure this scarce pre-hospital resource is available to be used when and where it is of highest value. Such measures could include guidelines on inter-facility ambulance use in specific clinical settings (e.g., trauma), auditing of referring providers and institutions to identify variability and opportunities for targeted intervention, or regional regulations. Such measures might be best implemented by different stakeholders, including institutions, ambulance providers, or regional governments. In some cases, it may be necessary to modify existing guidelines, including those that may inadvertently pressure providers to refer lower-acuity trauma patients [32]. Once ambulances are freed from lower-acuity referral demand, clinical and geospatial dispatch algorithms might be employed to best allocate them among patients arriving from the scene [34].

### Limitations

Our study has several limitations. As a single-center cross-sectional assessment, our findings may not generalize to other settings, including to rural or district-level hospitals, private facilities, and those that are not regional referral or specialty centers of excellence. Moreover, the high rates of referral among RTC patients and the relative abundance of ambulances we observed may limit replicability where pre-hospital and emergency health systems are organized differently or at different stages of development.

Because we employed ED triage records, we are inherently constrained in the inferences we are able to draw. Specifically, we are unable to draw conclusions regarding specific types of injuries, treatments obtained, clinical outcomes, or costs of care. In addition, we are unable to examine reasons underlying ambulance use decisions among providers or patients/laypersons, nor can we deduce appropriateness of referral decision in the first place. Several of these questions might be best explored through future survey-based and qualitative designs [35]. Nevertheless, our findings suggest considerable opportunities for improvement in local regional trauma system coordination, and highlight the utility of ED triage data as a feasible, low-cost, and efficient means of monitoring local pre-hospital use and referral patterns.

## Conclusions

As urban trauma and pre-hospital care systems mature across sub-Saharan Africa, regional governments and health policymakers should regularly and critically evaluate whether limited resources are being directed to those patients most likely to benefit. In Addis Ababa, ambulance use does not appear to parallel clinical acuity among patients presenting after RTC. Further studies should explore ambulance use in other contexts, as well as factors influencing ambulance selection for low-acuity patients. Reducing ambulance use among low-acuity inter-facility referrals may represent a targetable opportunity to increase ambulance value where they are scarcest.

## Additional files

Additional file 1: **Table S1.** Factors associated with ambulance arrival among RTC patients referred from another institution, AaBET Hospital, Addis Ababa. (DOCX 15 kb)

Additional file 2: **Table S2.** Factors associated with ambulance arrival among RTC patients arriving from the scene, AaBET Hospital, Addis Ababa. (DOCX 14 kb)

## Abbreviations

AaBET: Addis Ababa Burn Emergency and Trauma (Hospital)
aOR: Adjusted odds ratio
CI: Confidence interval
ED: Emergency department
ICU: Intensive care unit
LMIC: Low- and middle-income
RTC: Road traffic collisions
SATS: South Africa triage scale
SDGs: Sustainable development goals
SPHMMC: St. Paul’s Hospital Millennium Medical College
SPSS: Statistical Package for the Social Sciences
