# Supplementary material for: Ambulance use is not associated with patient acuity after road traffic collisions: a cross-sectional study from Addis Ababa, Ethiopia
Source: BMC Emerg Med. 2018 Feb 13;18:7. doi: 10.1186/s12873-018-0158-5 (PMC5810000; doi:10.1186/s12873-018-0158-5)
Supplement: Supplementary file 1 — Table S1. Factors associated with ambulance arrival among RTC patients referred from another institution, AaBET Hospital, Addis Ababa. (DOCX 15 kb) [file 12873_2018_158_MOESM1_ESM.docx]

**Additional file 1: Table S1** Factors associated with ambulance arrival among RTC patients referred from another institution, AaBET Hospital, Addis Ababa.

| **Characteristic** | **Arrival by ambulance** | | **Adjusted Odds of Ambulance Use** |
| --- | --- | --- | --- |
|  | **Yes (266)** | **No (115)** |  |
|  | *N (%)^1^* | *N (%)^1^* | *aOR (95%CI)* |
| Patient sex |  |  |  |
| Female | 93 (35.0) | 37 (32.2) | 1.0 *(ref)* |
| Male | 173 (65.0) | 78 (67.8) | 0.93 (0.57, 1.51) |
| Patient age |  |  |  |
| <13 | 19 (7.2) | 4 (3.5) | 1.85 (0.55, 6.22) |
| 13-24 | 74 (28.1) | 38 (33.3) | 1.14 (0.62, 2.11) |
| 25-40 | 110 (41.8) | 41 (36.0) | 1.33 (0.74, 2.39) |
| >40 | 60 (22.8) | 31 (27.2) | 1.0 *(ref)* |
| Patient origin |  |  |  |
| Addis Ababa | 72 (27.5) | 29 (25.2) | 1.10 (0.65, 1.87) |
| Outside Addis | 190 (72.5) | 86 (74.8) | 1.0 *(ref)* |
| Date of Arrival |  |  |  |
| Weekday | 183 (70.9) | 87 (77.0) | 0.74 (0.43, 1.26) |
| Weekend | 75 (29.1) | 26 (23.0) | 1.0 *(ref)* |
| Triage Acuity*^2,3^* |  |  |  |
| Low Acuity | 103 (40.2) | 49 (43.0) | 1.0 *(ref)* |
| Moderate Acuity | 109 (42.6) | 53 (46.5) | 0.96 (0.58, 1.57) |
| High Acuity | 44 (17.2) | 12 (10.5) | 1.57 (0.73, 3.42) |
| Referral status/source |  |  |  |
| Referred from government hospital | 191 (71.8) | 55 (47.8) | 4.39 (1.53, 12.63) |
| Referred from health center | 68 (25.6) | 51 (44.3) | 1.79 (0.60, 5.31) |
| Referred from private institution | 7 (2.6) | 9 (7.8) | 1.0 *(ref)* |

^1^ Percent of non-missing data reported.

^2^ South Africa Triage Scale acuity designations.

^3^ High acuity includes very urgent, emergent, and dead on arrival. Dead on arrival grouped within very urgent or emergent due to presumed scene/pre-hospital acuity.
