# Supplementary material for: Ambulance use is not associated with patient acuity after road traffic collisions: a cross-sectional study from Addis Ababa, Ethiopia
Source: BMC Emerg Med. 2018 Feb 13;18:7. doi: 10.1186/s12873-018-0158-5 (PMC5810000; doi:10.1186/s12873-018-0158-5)
Supplement: Supplementary file 2 — Table S2. Factors associated with ambulance arrival among RTC patients arriving from the scene, AaBET Hospital, Addis Ababa. (DOCX 14 kb) [file 12873_2018_158_MOESM2_ESM.docx]

**Additional file 2: Table S2** Factors associated with ambulance arrival among RTC patients arriving from the scene, AaBET Hospital, Addis Ababa.

| **Characteristic** | **Arrival by ambulance** | | **Adjusted Odds of Ambulance Use** |
| --- | --- | --- | --- |
|  | **Yes (84)** | **No (115)** |  |
|  | *N (%)^1^* | *N (%)^1^* | *aOR (95%CI)* |
| Patient sex |  |  |  |
| Female | 43 (51.2) | 33 (28.7) | 1.0 *(ref)* |
| Male | 41 (48.8) | 82 (71.3) | 0.37 (0.19, 0.72) |
| Patient age |  |  |  |
| <13 | 0 (0) | 8 (7.1) | N/A^4^ |
| 13-24 | 36 (43.9) | 37 (33.0) | 1.71 (0.71, 4.11) |
| 25-40 | 29 (35.4) | 42 (37.5) | 1.27 (0.53, 3.04) |
| >40 | 17 (20.7) | 25 (22.3) | 1.0 *(ref)* |
| Patient origin |  |  |  |
| Addis Ababa | 47 (56.0) | 83 (72.2) | 0.50 (0.26,0.99) |
| Outside Addis | 37 (44.0) | 32 (27.8) | 1.0 *(ref)* |
| Date of Arrival |  |  |  |
| Weekday | 62 (76.5) | 69 (61.1) | 2.02 (0.99, 4.14) |
| Weekend | 19 (23.5) | 44 (38.9) | 1.0 *(ref)* |
| Triage Acuity*^2,3^* |  |  |  |
| Low Acuity | 37 (45.7) | 75 (65.2) | 1.0 *(ref)* |
| Moderate Acuity | 38 (46.9) | 32 (27.8) | 2.97 (1.50, 5.86) |
| High Acuity | 6 (7.4) | 8 (7.0) | 1.67 (0.48, 5.82) |

^1^ Percent of non-missing data reported.

^2^ South Africa Triage Scale acuity designations.

^3^ High acuity includes very urgent, emergent, and dead on arrival. Dead on arrival grouped within very urgent or emergent due to presumed scene/pre-hospital acuity.

^4^ There were no patients less than thirteen years of age brought by ambulance from the scene.
